# Breast implant-associated anaplastic large cell lymphoma: a case report and literature review

**DOI:** 10.3389/fonc.2026.1889467

**Published:** 2026-08-06

**Authors:** Shuangyi Qi, Hu Liu, Qidi Huang, Huayan Gu, JiaLing Ding, Guilong Guo, Shurong Zheng

**Affiliations:** 1 Department of Breast Surgery, First Affiliated Hospital of Wenzhou Medical University, Wenzhou, China; 2 Department of General Surgery, Taishun County People’s Hospital, Wenzhou, China

**Keywords:** BIA-ALCL, breast implant, diagnosis process, pathological examination, textured implant

## Abstract

Breast implant-associated anaplastic large cell lymphoma (BIA-ALCL) is a rare T-cell non-Hodgkin lymphoma primarily associated with textured breast implants. Its pathological hallmarks include CD30 positivity, ALK negativity, and clonal T-cell receptor gene rearrangement. Complete surgical excision is the primary treatment modality, and early diagnosis combined with standardized management is associated with a favorable prognosis. We report a case of BIA-ALCL in a 47-year-old female who presented with breast swelling 20 years after receiving textured breast implants. To the best of our knowledge, this is the first documented case of BIA-ALCL in mainland China. Preoperative aspiration was not suggestive of BIA-ALCL, and the diagnosis was ultimately confirmed through histopathological examination of the capsule tissue. Based on a comprehensive postoperative systemic evaluation, no adjuvant therapy was required.

## Introduction

Breast implant-associated anaplastic large cell lymphoma (BIA-ALCL) is a rare T-cell non-Hodgkin lymphoma (1). Keech and Creech first reported this in 1997, and subsequent studies confirmed its association with textured-surface breast implants (2–4). In 2016, the World Health Organization (WHO) called it a new type of ALK-negative lymphoma (5). Then, in 2017, the National Comprehensive Cancer Network (NCCN) issued guidelines for this disease (6). The 2022 WHO Classification of Hematolymphoid Tumors (5th edition) and the International Consensus Classification (ICC) have now recognized BIA-ALCL as a distinct diagnostic entity (7, 8). Statistical data show that up to May 30, 2025, the American Society of Plastic Surgeons has recorded 1,618 cases of BIA-ALCL globally, with 458 of them being suspected or confirmed cases in the United States (9).

The most common symptoms of BIA-ALCL are seroma (periprosthetic fluid accumulation) or a solid mass occurring more than one year after implant placement, while additional symptoms include breast swelling, pain, cutaneous manifestations (such as inflammation and rash), and axillary lymphadenopathy (10–12). As a T-cell lymphoma, its main microscopic features are clusters of abnormal T cells (13). Nearly all cases test positive for CD30, while other key characteristics include ALK negativity and clonal T-cell receptor gene rearrangement (14–17). Complete surgical excision, as the main treatment for BIA-ALCL, helps significantly reduce the risk of recurrence and improve patient survival rates (10, 18, 19). Overall, the prognosis of this disease is relatively good with early diagnosis and standardized treatment (18).

To the best of our knowledge, no case of BIA-ALCL has been documented in mainland China to date. We present the first recorded case, in a patient who underwent implant removal with complete capsulectomy and a comprehensive staging workup. This report adheres to the CARE guidelines for case reporting.

## Case presentation

The patient was a 47-year-old Chinese female who presented in December 2025 with complaints of bilateral breast asymmetry, as well as swelling accompanied by pruritus and pain in the right breast. She had undergone bilateral breast augmentation with textured implants in Wenzhou, China, more than 20 years prior to presentation. She had no significant past medical history and no family history of breast cancer or lymphoma. Ultrasound revealed periprosthetic fluid accumulation surrounding the right implant.

**Figure 1:**
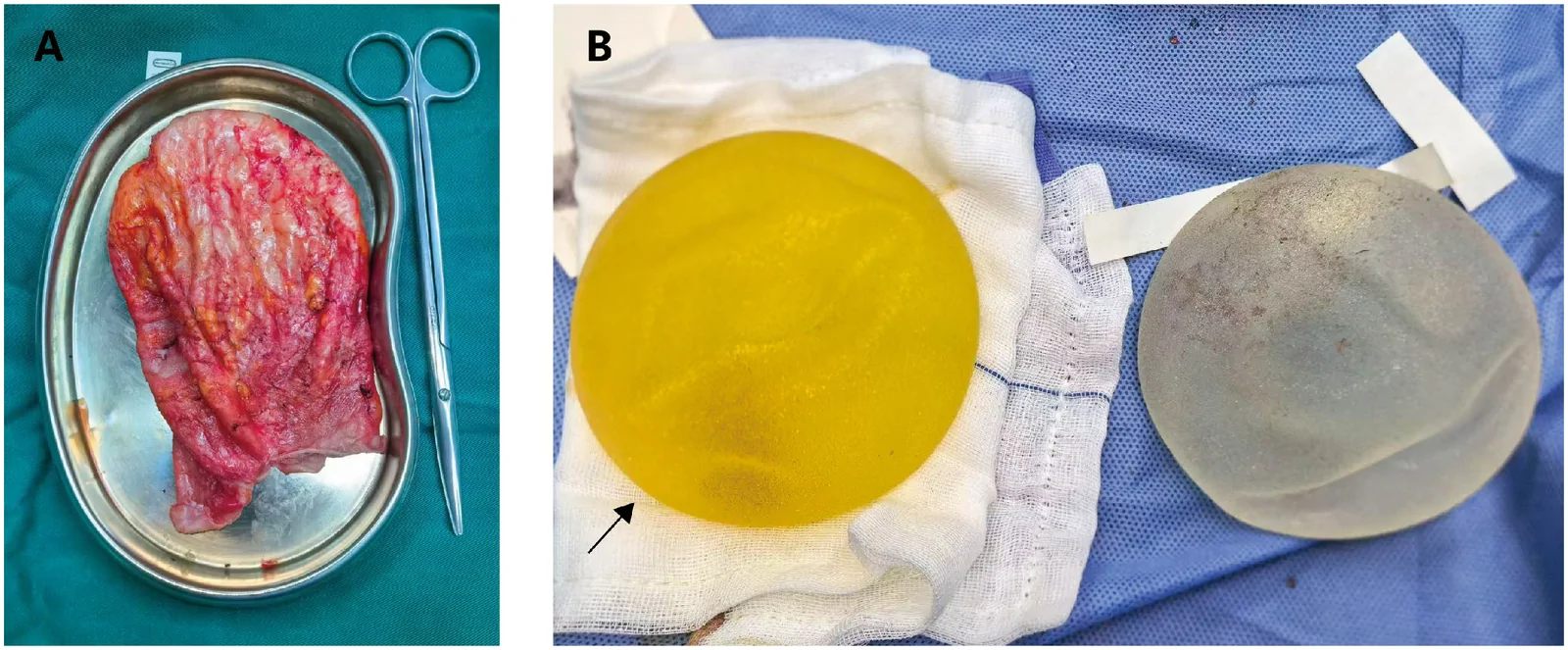
**(A)** Right implant capsule tissue. **(B)** Breast prosthesis, the arrow indicates the right side.

**Figure 2:**
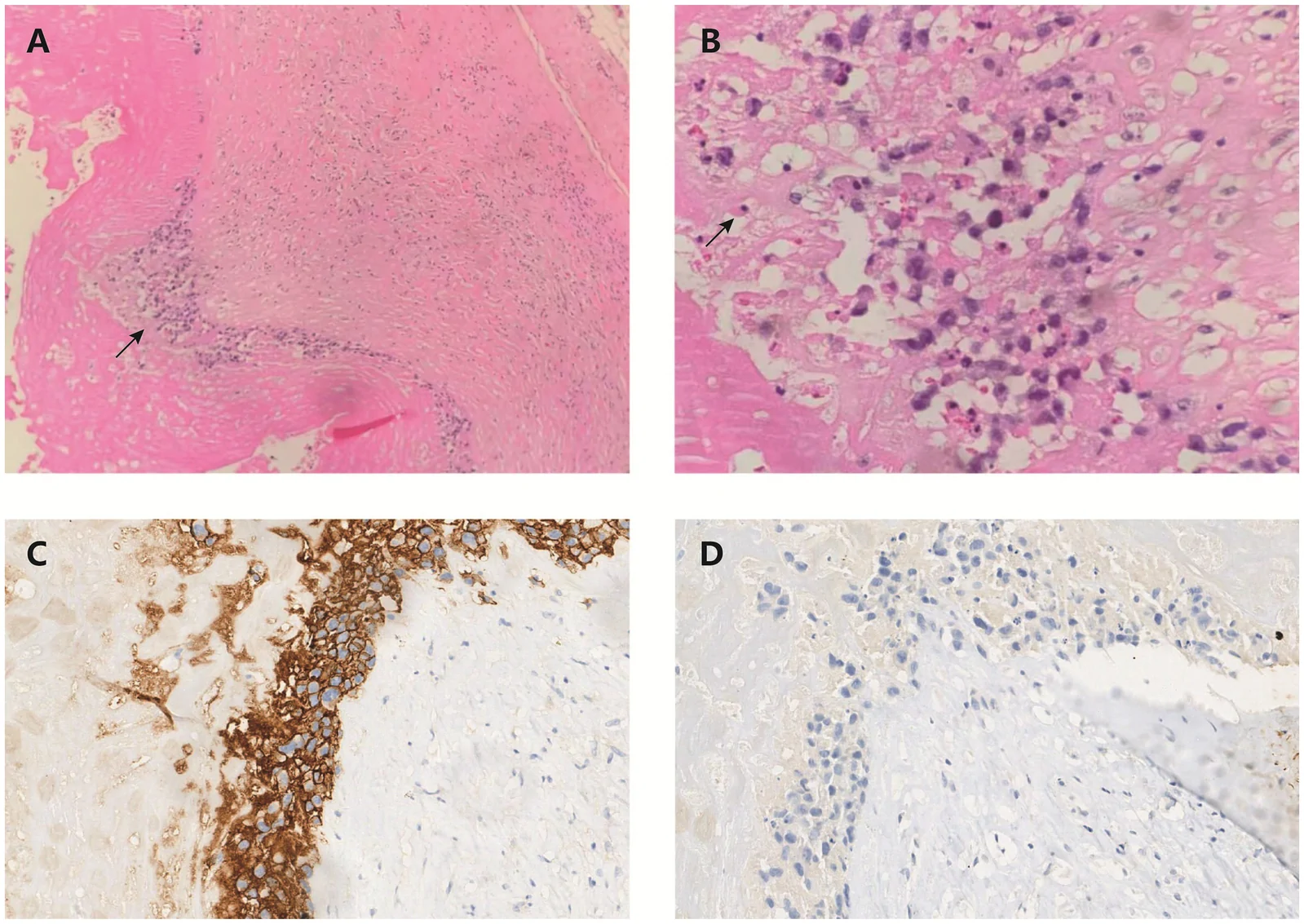
**(A)** A bland fibrous capsule is identified, with occasional reactive lymphoid aggregates (arrow) (H&E, ×10). **(B)** The stroma demonstrates lymphocytic and eosinophilic infiltration, accompanied by scattered atypical cells with large, hyperchromatic nuclei (H&E, ×40). **(C)** Immunohistochemical staining shows CD30 at the luminal side, consistent with pathologic stage T1 (IHC, ×20). **(D)** Immunohistochemical staining for ALK is negative in the atypical cells (IHC, ×20).

Under ultrasound guidance, we aspirated the periprosthetic fluid from the right side and prepared a cell smear; pathological examination revealed blood components and scattered degenerated inflammatory cells. The patient subsequently underwent bilateral breast implant removal and capsulectomy via a periareolar incision. Intraoperatively, both implants were observed to be located in the retromammary space. The surface of the right implant appeared yellowish, and approximately 300 ml of fluid was found within the capsule, containing flocculent yellow debris (**Figure 1**). Postoperative immunohistochemical analysis was performed on the capsular tissues of both breast implants. The results demonstrated that the atypical lymphoid cells in the right implant capsule tissue showed positivity for CD30, CD4, and EMA, and negativity for ALK, with no evidence of extracapsular extension, findings consistent with the pathological diagnosis of BIA-ALCL (**Figure 2**). On the left side, no definitive malignancy was identified. All surgical margins were negative postoperatively. The final diagnosis was reviewed and confirmed by an experienced hematopathologist at our institution.

**Figure 3:**
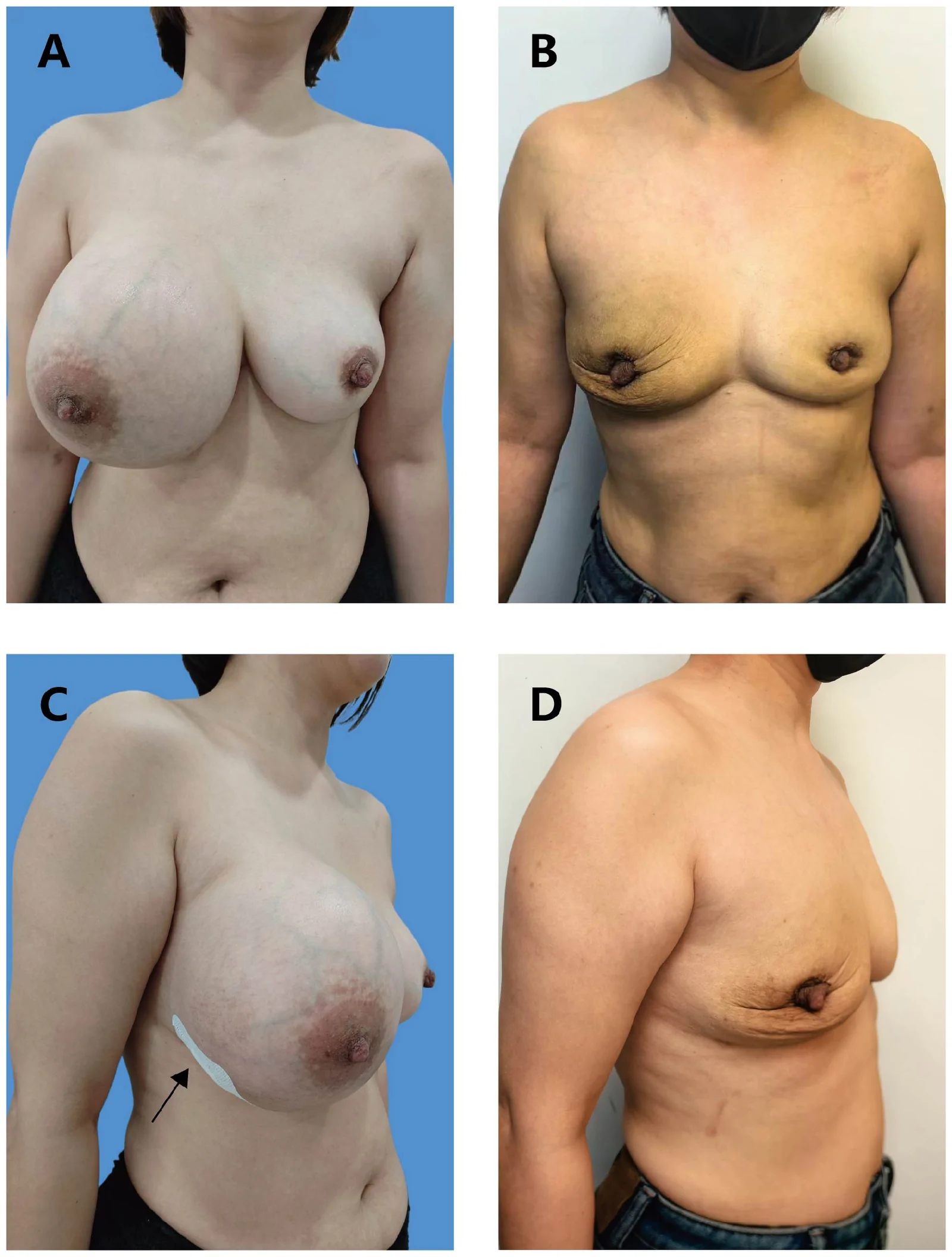
**(A)** Preoperative frontal view. **(B)** Postoperative frontal view. **(C)** Preoperative three-quarter view. The arrow indicates the dressing covering the puncture site. **(D)** Postoperative three-quarter view.

**Figure 4:**
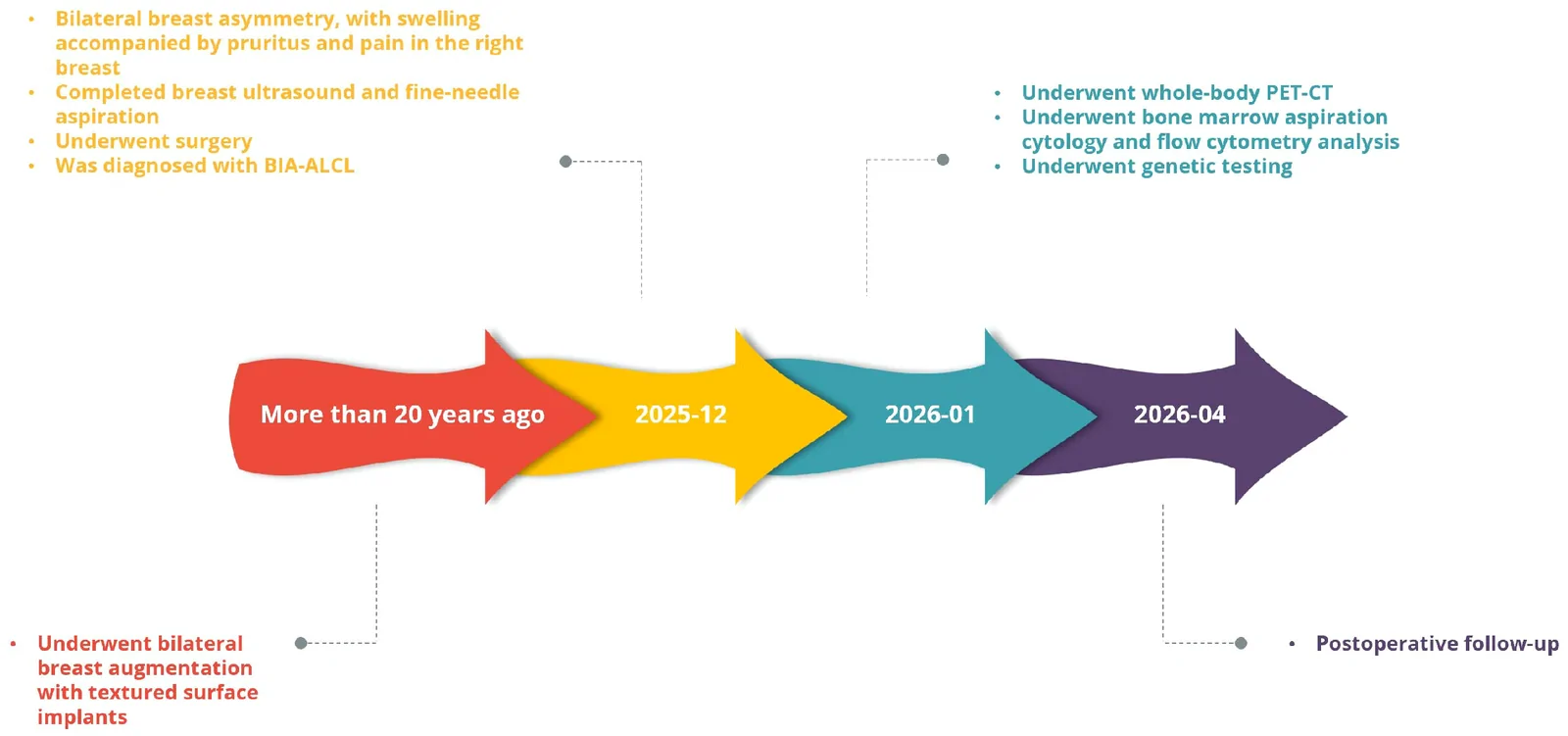
Diagnostic and treatment process.

Postoperatively, the patient underwent whole-body PET-CT, which revealed that the lesion was confined to the local breast area, with no evidence of lymph node or distant organ involvement. According to the NCCN guidelines, the clinical stage was determined to be IA (T1N0M0). The patient subsequently underwent bone marrow biopsy. Pathological examination of the bone marrow specimen showed no tumor involvement. Flow cytometric analysis performed on the same bone marrow aspirate demonstrated positivity for CD5, CD58, CD2, CD3, CD7, CD8, and CD4, and negativity for CD11c, HLA-DR, CD56, and CD16. Genetic testing on the bone marrow sample indicated that the patient was negative for B-cell lymphoma gene rearrangement and for T-cell receptor (TCR) gene rearrangement. Based on the staging and comprehensive assessment results, the patient did not require adjuvant chemotherapy or radiotherapy. At the three-month postoperative follow-up, physical examination revealed normal vital signs (**Figure 3**), and breast ultrasound revealed no new breast lesions. The patient reported satisfactory cosmetic results and no functional impairment. The diagnostic and treatment process is shown in **Figure 4**.

## Discussion

BIA-ALCL is a rare complication associated with breast implants. According to a systematic review, there is a long latency period between implant placement and the onset of BIA-ALCL, averaging approximately 11 years, with about 81% of cases being diagnosed within this time frame (18). The reported incidence rates in the literature vary considerably, with the proportion of affected patients ranging from 1 in 3,500 to 1 in 86,029, and incidence rates ranging from 1 per 3,215 to 1 per 500,000 person-years (20–23). However, in specific high-risk subgroups with textured implants, the risk has been reported to be as high as 1 in 355 (24). This substantial discrepancy may stem from significant limitations faced by epidemiological studies: the limited number of confirmed cases worldwide, coupled with the difficulty in accurately quantifying implant sales data, the number of implants currently *in situ*, and their distribution by brand, makes it challenging to accurately assess the true risk of developing this disease.

As of June 30, 2024, among the 1,050 case reports submitted to the U.S. Food and Drug Administration (FDA) where the implant surface type was specified, nearly all patients (1,010/1,050, 96.2%) had received textured implants, indicating a strong association between the development of BIA-ALCL and this implant type (25). The pathogenesis of BIA-ALCL has not yet been fully elucidated. The prevailing hypothesis suggests that the textured surface may act as a chronic irritant, persistently inciting a local immune response. This promotes the chemotaxis and accumulation of inflammatory cells, such as CD4+ T lymphocytes, thereby creating a microenvironment conducive to lymphomagenesis (26, 27). An alternative hypothesis proposes that the textured surface may be more prone to bacterial colonization and biofilm formation, and the resulting chronic inflammation could similarly drive the abnormal proliferation of lymphocytes (28). At the molecular level, studies have frequently identified mutations in genes such as JAK/STAT, TP53, and DNMT3A within BIA-ALCL tumor cells. This suggests that alterations in these signaling pathways are crucial events in the disease’s development; however, the specific mechanisms of their action require further investigation (29, 30).

The clinical presentation of BIA-ALCL is typically indolent and can be categorized into two types: seroma-predominant and mass-forming (31, 32). It is important to note that not all delayed-onset seromas are associated with BIA-ALCL; only approximately 9% are ultimately diagnosed as such (33). For highly suspicious cases, the following examinations are recommended to confirm the diagnosis: for imaging, ultrasound should be the preferred method due to its safety, non-invasive nature, and reproducibility (34); MRI is recommended when ultrasound fails to clearly visualize the fluid collection or mass. PET-CT may be performed to comprehensively assess disease stage, detect metastatic sites, and guide lymph node biopsy (35, 36). Definitive diagnosis relies on pathological examination: specimens are typically obtained via fine-needle aspiration of periprosthetic fluid. Immunohistochemical staining typically reveals positivity for CD30 and EMA, and negativity for ALK; molecular testing demonstrates evidence of clonal T-cell receptor (TCR) gene rearrangement (13, 15, 17).

However, in the present case, the aspiration smear did not yield a definitive diagnosis of BIA-ALCL, likely because the diagnosis depends on comprehensive analysis of both cellular morphology and immunophenotype (37), and a simple fluid aspiration smear has limitations in both respects. BIA-ALCL tumor cells in the fluid may be present in very low numbers and sparsely distributed, and are often admixed with blood components, fibrin, and reactive inflammatory cells. This makes fine-needle aspiration samples prone to sampling error, as tumor cells may be lost during aspiration or slide preparation. Furthermore, under routine cytological staining, the morphology of degenerated inflammatory cells and reactive lymphocytes may resemble that of low-grade lymphoma cells. In the absence of typical clusters of highly atypical large lymphocytes, pathologists often find it difficult to make a direct diagnosis of malignancy. The key diagnostic marker for confirming BIA-ALCL is CD30 positivity, and it is necessary to rule out other lymphomas (such as ALK-positive anaplastic large cell lymphoma) through immunohistochemical staining (14, 37). Immunohistochemistry must be performed on cell blocks or tissue sections, as reliable multi-marker immunophenotypic analysis cannot be achieved with cell smears alone. Therefore, the diagnosis of BIA-ALCL should not rely solely on cell smears, and a negative result from an initial aspiration cannot rule out the disease.

Earlier studies have suggested that BIA-ALCL tumor cells originate from an early precursor cell in which T-cell receptor (TCR) rearrangement has not yet been initiated or completed. During neoplastic transformation, although the cells acquire a malignant phenotype, their differentiation program may become arrested prior to TCR gene rearrangement, or they may actively downregulate TCR expression during subsequent oncogenesis. This results in the final tumor cells lacking clonal TCR gene rearrangement (38). Molecular analysis of BIA-ALCL can reveal monoclonal T-cell receptor gamma gene rearrangement, although a subset of systemic ALCL does not exhibit T-cell receptor clonality (39, 40). Although TCR gene rearrangement was negative in this case, differential diagnosis among reactive CD30+ effusion, systemic ALK-negative ALCL, and BIA-ALCL is required. Reactive CD30+ T cells in periprosthetic seroma typically exhibit a polyclonal TCR pattern or reactive oligoclonal expansion within a mixed inflammatory background (which may present as acute, mixed, or chronic inflammation with neutrophils, lymphocytes, and macrophages), along with an extremely low proportion of CD30+ cells (<5%) and the absence of cytologic atypia (15, 40). In contrast, our case demonstrated numerous atypical cells within the right implant capsule, with strong CD30 expression and absence of CD3/ALK, confined to the periprosthetic capsule and without systemic involvement. Systemic ALK-negative ALCL typically presents with generalized lymphadenopathy or extranodal involvement (skin, soft tissue, liver, spleen, and elsewhere), often at stage III-IV with B symptoms (41). Therefore, despite the negative TCR result, the diagnosis of BIA-ALCL remains well supported. Nevertheless, the reason for the negative TCR gene rearrangement in our patient still warrants discussion.

Complete surgical excision is the primary treatment modality for BIA-ALCL (42). Compared to partial capsulectomy alone, systemic chemotherapy, or radiotherapy, complete surgical excision significantly improves both overall survival (P = 0.022) and event-free survival (P = 0.014) (10). The extent of surgery should include the implants, the surrounding capsule, any associated mass and fluid collection, as well as any region potentially involved by the tumor, especially lymph nodes (43). Lymph node involvement is a critical prognostic factor, often indicating disease progression or a more clinically aggressive course: the survival rate for patients without lymph node metastasis is 97.9%, whereas with metastasis, the survival rate drops significantly to 75% (44). Furthermore, approximately 2% to 4% of patients may have contralateral involvement; therefore, surgeons should assess the need for simultaneous removal of the contralateral implant and capsule (45). Breast reconstruction may be considered postoperatively; available options include reconstruction with smooth-surface implants, immediate mastopexy, autologous tissue reconstruction, and fat grafting (42). For advanced-stage cases, adjuvant therapies such as radiotherapy, chemotherapy, and targeted therapy are indicated (43, 46). As the patient in this case had no future requirements for fertility or breastfeeding, the periareolar approach was selected for the bilateral breast implant removal and capsulectomy. This incision, located at the border of the areola, results in inconspicuous postoperative scarring and offers a favorable aesthetic outcome. Owing to the patient’s large areola and good skin elasticity, adequate exposure was achieved, allowing the procedure to be performed under direct visualization with a clear surgical field, thereby facilitating precise dissection and manipulation for complete excision.

Genetic markers may hold prognostic value in BIA-ALCL and could serve as future therapeutic targets; however, the current evidence regarding the prognostic significance of such genetic testing remains limited (46). Genomic studies have shown that patients with BIA-ALCL typically exhibit an accumulation of chromosomal and genetic abnormalities above average (47). Reports from the Danish national registry have suggested that germline BRCA1/2 mutations may increase the risk of developing BIA-ALCL, with an estimated risk of 1 in 1,550 (48). Patients carrying BRCA1/2 or TP53 gene mutations have significantly shorter event-free survival times, at 115 months and 36 months respectively, compared to 148 months for patients without these mutations (22). Therefore, the presence of genetic susceptibility factors such as BRCA or TP53 mutations may constitute an additional contraindication for the use of textured surface implants.

There are several limitations to this case report. First, since the primary surgery was performed more than 20 years ago and no mandatory medical device traceability system existed in China at that time, the implant manufacturer and model could not be identified. This limitation prevented a precise description of surface texture grading and risk stratification. Second, the negative result of the TCR gene rearrangement assay limited the diagnostic utility of clonality testing in this case. Third, the immunophenotypic results from the primary lesion and bone marrow were derived from different specimens. On the capsulectomy specimen, immunohistochemistry showed diffuse, strong CD30 positivity and absence of ALK and CD3 expression, findings consistent with the characteristic immunophenotype of BIA-ALCL (12). In the bone marrow, flow cytometry demonstrated a polyclonal T-cell population positive for CD3, CD2, CD5, CD7, CD4, and CD8, with no clonal expansion or aberrant immunophenotype, thereby excluding bone marrow involvement. These two findings are not contradictory, reflecting respectively T-cell antigen loss in the tumor cells and the presence of reactive background T cells in the bone marrow (37). Despite these limitations, the diagnosis of BIA-ALCL was firmly established through the integration of clinical history, characteristic histomorphology (hallmark cells), and supportive immunohistochemistry (CD30+/ALK−), with confirmation by an experienced hematopathologist.

## Data Availability

The original contributions presented in the study are included in the article/supplementary material. Further inquiries can be directed to the corresponding authors.
